# Supplementary material for: Dendro-dendritic cholinergic excitation controls dendritic spike initiation in retinal ganglion cells
Source: Nat Commun. 2017 Jun 7;8:15683. doi: 10.1038/ncomms15683 (PMC5477517; doi:10.1038/ncomms15683)
Supplement: Supplementary Information — Supplementary Figures and Supplementary Methods [file ncomms15683-s1.pdf]

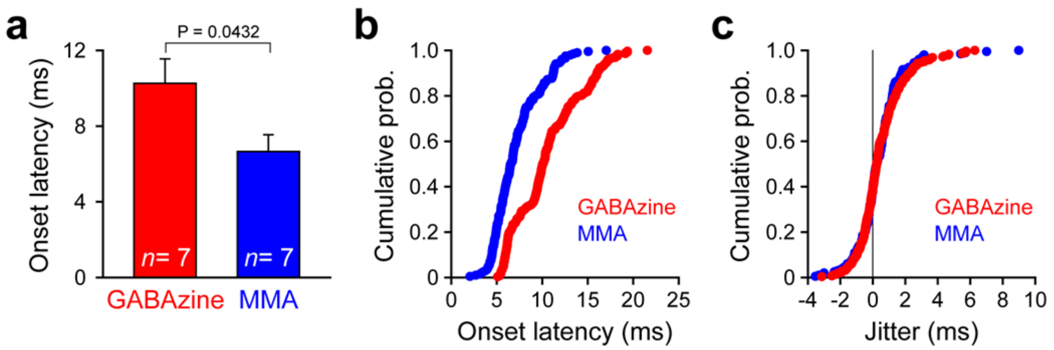

Supplementary Figure 1 Onset latency of SAC-evoked excitatory and inhibitory postsynaptic potentials. (**a**) Average onset latency of pharmacologically isolated ON-SAC-evoked excitatory and inhibitory postsynaptic potentials recorded in the presence of GABAzine (10 µM, red) and mecamylamine (MMA; 10 µM, blue), respectively. (**b**) Cumulative probability distributions of the onset latency of ON-SAC-evoked PSPs recorded under the indicated conditions. (**c**) Cumulative probability distribution of the temporal jitter of the onset latency of individual ON-SAC-evoked PSPs with respect to the mean response averaged across at least 26 trials. Data has been collated form pairs recorded in the presence GABAzine (10 µM, red, *n*= 7) and mecamylamine (MMA; 10 µM, blue, *n*= 7).

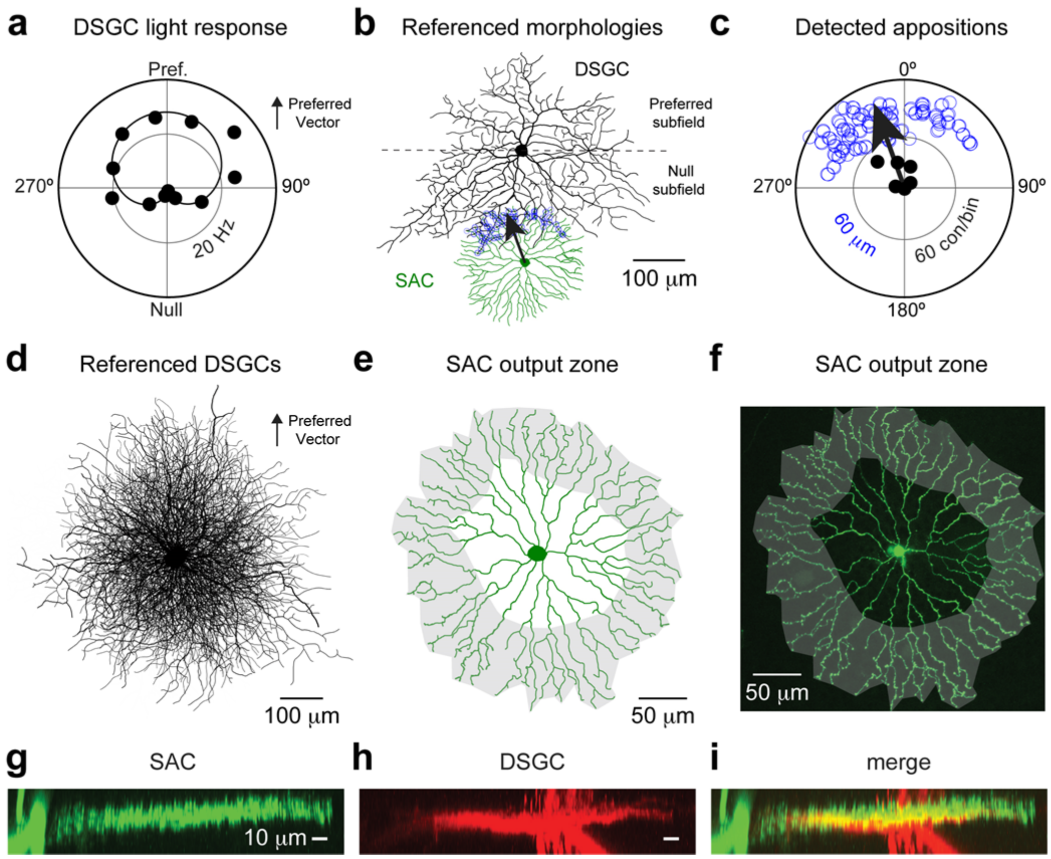

Supplementary Figure 2 Analysis methods used to estimate the position of SAC-DSGC dendro-dendritic close appositions. (**a**) Polar plot of moving light bar-evoked ON-DSGC action potential firing. The relationship has been fit with a sine function and a preferred direction vector calculated by vector summation. The plot has been rotated to point the vector north. Note that preferred direction light stimuli move in a direction 180° rotated to the preferred direction vector of action potential output. (**b**) Reconstruction of a simultaneously recorded ON-DSGC (same cell as panel a) and a null side ON-SAC. The morphologies have been aligned to the preferred-direction vector. Dendro-dendritic appositions between the terminal one third of each SAC dendritic branch (see panel e) and DSGC dendrites were automatically detected when dendrites were apposed by <= 0.15 µm for 1.5 µm. The sites (open circles) and vectorial angle of close dendro-dendritic appositions are shown. (**c**) Polar representation of the dendro-dendritic appositions shown in panel b. The blue symbols show appositions in distance coordinates from SAC soma, and the black symbols show the number of appositions per 30°

bin. The arrow represents vectorial summation of binned data. (**d**) Soma aligned, reconstructed ON-DSGCs that have been rotated according to the functionally determined preferred direction vector. A spatially filtered version of this image is shown in **Fig. 2e**. (**e**) Reconstruction of a SAC; the shaded grey area delineates the distal one third of the dendritic tree determined for each dendritic branch by measuring path lengths. (**f**) A confocal stack of the same SAC shows that this dendritic area is decorated by varicosities and is considered to represent the synaptic release, or output, zone. (**g-i**) Z-plane projected confocal image of a simultaneously recorded ON-SAC and ON-DSGC. Note the prominent dendritic co-fasciculation.

Supplemental Methods

The software used to perform close apposition analysis of the spatially overlapping dendritic arbors of DSGC and SAC neurons was developed as a package in MATLAB and is available online as open-source software at http://github.com/QBI-software/Neurites. The software uses boundary analysis from the MATLAB Image Processing Toolbox to detect intersecting dendritic regions within a determined region of interest. Data from Neurolucida measurements of reconstructed neurons were used to populate metadata of the dendrites and used in subsequent analysis of the relative positions of close dendro-dendritic appositions. Matching of boundary segments was performed within configurable pixel limits and duplication of regions was detected via a configurable parameter. The software enabled visualisation of the location of dendritic appositions on original images with reference to the relevant dendritic branches of either the DSGC or SAC somata.

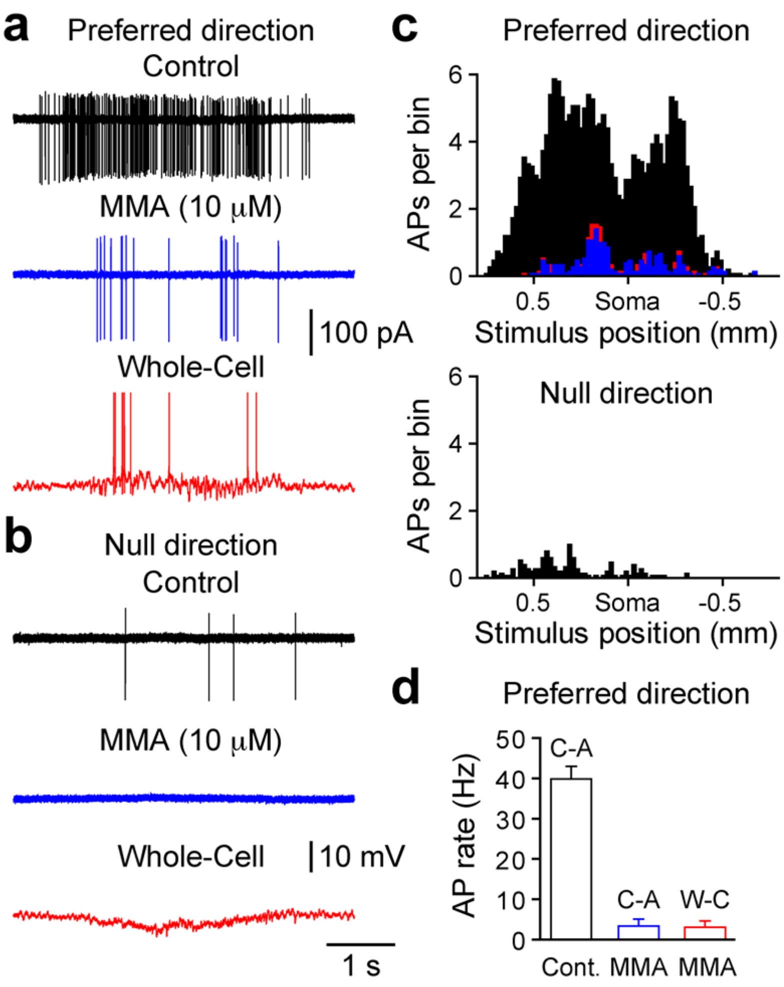

Supplementary Figure 3 Cholinergic control of preferred and null direction light responses is not perturbed by whole-cell recording techniques. (**a**) Non-invasive cell-attached patch recording of the powerful reduction of preferred direction light bar-evoked action potential firing by the nAChR antagonist mecamylamine (MMA; 10 μM, blue trace). The lower red trace shows the pattern of AP firing in the same neuron when the whole-cell recording configuration was subsequently obtained. (**b**) Non-invasive recording of the silencing of null direction light bar evoked AP firing by MMA. (**c**) Peri-stimulus histogram of the reduction of preferred (upper graph) and null direction (lower graph) AP firing recorded in cell-attached patches (black bars control, blue bars MMA), and subsequently under whole-cell recording conditions (red bars MMA; data pooled from 5 recordings; bin size 20 μm). (**d**) Pooled data showing the reduction of preferred direction AP firing rate by the antagonism of nAChRs in cell-attached recordings. The red bar illustrates the firing rate

subsequently recorded in whole-cell mode (firing rate in cell-attached mode significantly different between conditions; $P<0.001$; q= 19.21; firing rate in MMA not significantly different between recording modes; q= 0.74; data represent mean ± s.e.m).

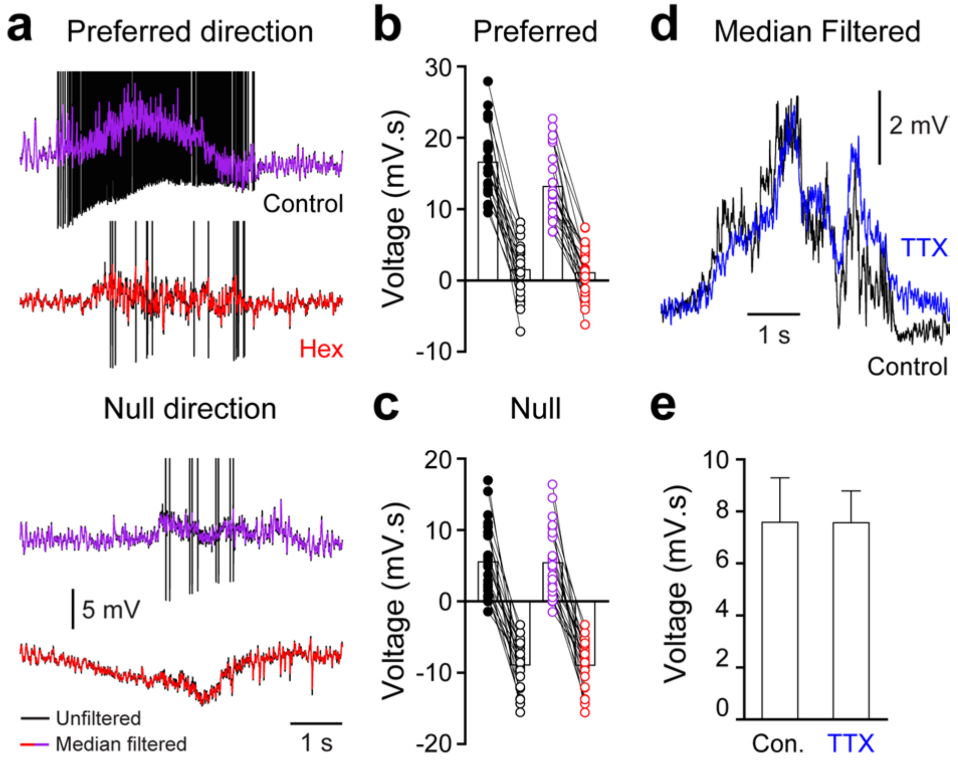

Supplementary Figure 4 Effects of median filtering on the integral of light responses. (**a**) Raw (black) and median filtered (coloured, 10 ms window) voltage records illustrate the reduction of preferred direction light responses, and the transformation of null direction responses by the antagonism of nAChRs (hexamethonium (Hex); 100 µM). Note that median filtering effectively removed action potentials from the voltage waveforms. (**b-c**) Quantification of the reduction of the integral of raw and median filtered preferred (b) and null (c) direction light responses by antagonism of nAChRs (colour code as in panel a). (**d-e**) Comparison of the integral of median filtered preferred direction light responses recorded in the absence (control) and presence of the sodium channel blocker TTX (*n*= 5, mean ± s.e.m).

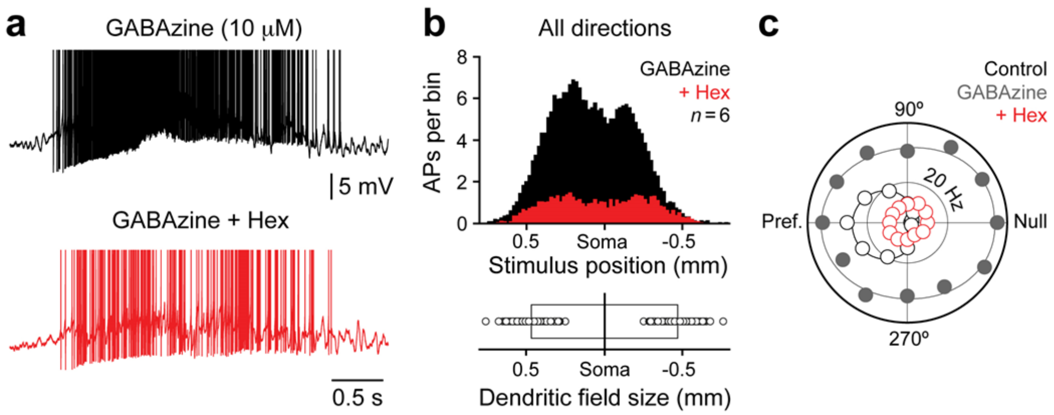

Supplementary Figure 5 Influence of cholinergic signalling on light-evoked action potential firing when GABAergic inhibition is blocked. (**a**) Antagonism of nAChRs (hexamethonium (Hex); 100 µM) attenuates moving bar light-evoked AP output when synaptic inhibition is blocked (GABAzine). (**b**) Peri-stimulus histogram of AP firing under the indicated conditions (upper graph; bin size 20 µm). The lower graph shows dendritic field size. (**c**) Polar plot of AP output under the indicated conditions, aligned to the control preferred direction (light intensity: control= 100%; GABAzine conditions= 38 ± 5 %).

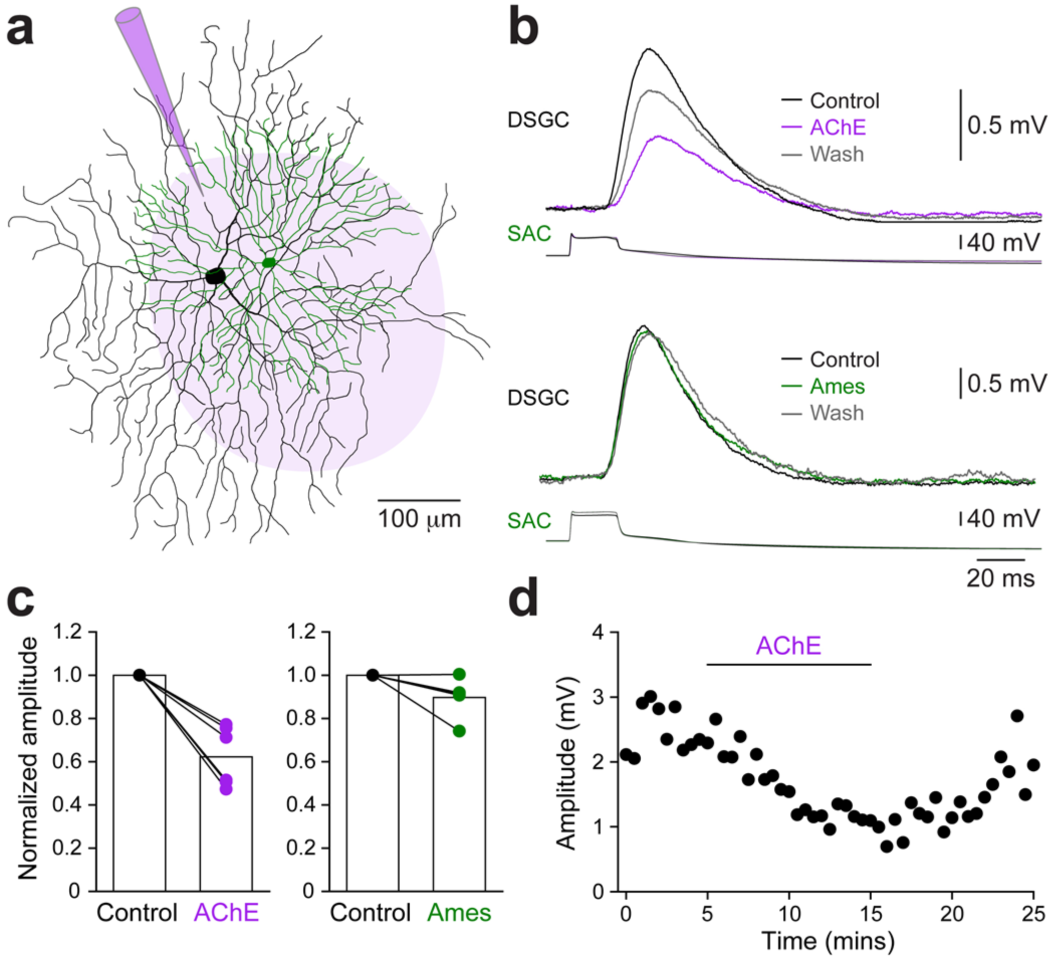

Supplementary Figure 6 SAC-evoked cholinergic excitation is attenuated by exogenous AChE. (**a**) Reconstruction of a connected ON-SAC-ON-DSGC pair showing schematically the placement of the local application pipette and the area of local drug delivery, determined visually by deformation of the IPL. (**b**) Representative traces showing the reversible attenuation of unitary SAC-evoked excitatory PSPs by the local pressure application of AChE (0.4 U per μl), and the lack of affect of the local pressure application of Ames solution. (**c**) Pooled data showing the attenuation of the amplitude of nAChR-mediated PSPs by exogenous AChE (left graph), but not the local application of Ames solution (right graph). (**d**) Representative example of the time course of the reversible reduction of unitary SAC-mediated excitation by exogenous AChE.

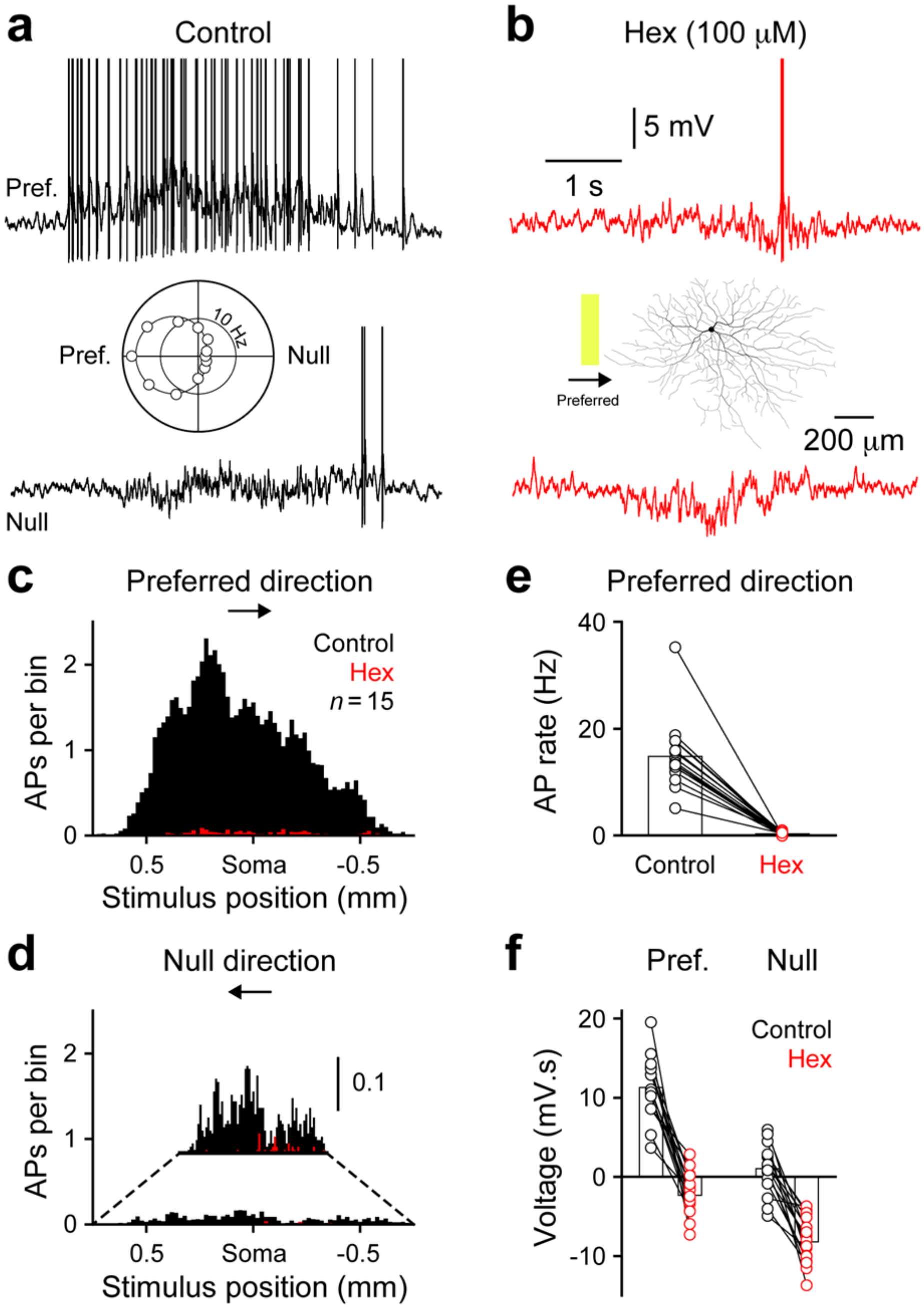

Supplementary Figure 7 Influence of cholinergic signalling on direction selective light responses under mesopic (100% stimulus contrast) conditions. (**a-b**) Preferred and null direction light responses evoked by dim light stimuli under control (a) and in the presence of hexamethonium (b; (Hex); 100 μM). The insets show a polar plot of action potential (AP) output, which has been fit with a sine function, and a reconstruction of the illustrated ON-DSGC. (**c-d**) Peri-stimulus histogram of the reduction of preferred (c) and null direction (d)

AP firing recorded under the indicated conditions (data pooled from 11 recordings; bin size 20 μm). (**e**) Quantification of the reduction of AP output evoked by preferred direction light stimuli when nAChRs were blocked. (f) Transformation of the voltage integral of median filtered preferred and null direction light responses by the blockade of nAChRs,

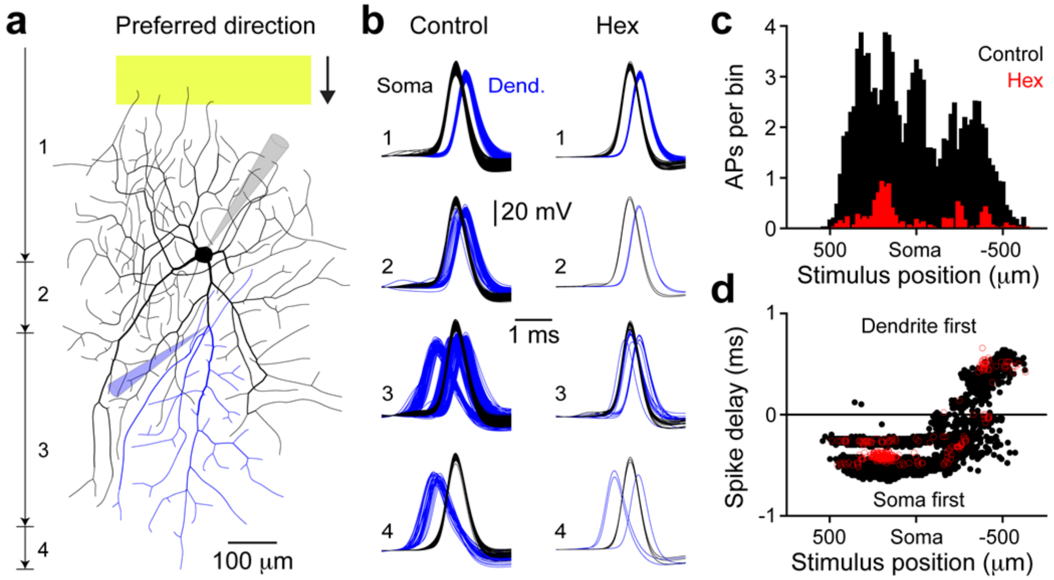

Supplementary Figure 8 Cholinergic signalling controls light-evoked dendritic spike initiation. (**a**) Reconstruction of an ON-DSGC showing the placement of somatic and null dendritic subfield recording electrodes, and the preferred direction movement of a light bar. The blue coloured section of the dendritic tree feeds to the dendritic recording site. (**b**) The generation of back-propagating action potentials (dendritic recording (blue), positions 1 and 2 in a), and dendritic spikes (positions 3 and 4 in a) were attenuated by antagonism of nAChRs (hexamethonium (Hex); 100 µM) when a preferred direction light bar was swept across the receptive field. Traces are aligned to the peak of somatically recorded action potentials (APs). (**c**) Summary of the reduction of AP output by hexamethonium. (**d**) Spatial pattern of somato-dendritic spike delay under control (black symbols) and following the blockade of nAChRs (Hex, red symbols).

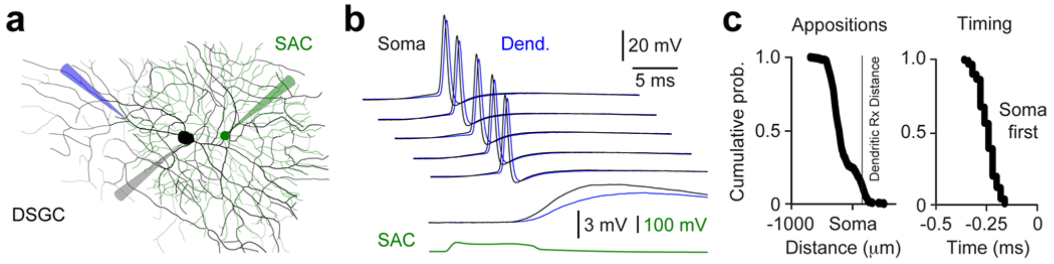

Supplementary Figure 9 Activation of a single SAC drives neuronal output. (**a**) Reconstruction of simultaneously recorded ON-SAC and ON-DSGCs, showing the placement of recording electrodes. Note that the dendritic recording electrode is positioned in the subtree contralateral to the SAC. (**b**) ON-DSGC somatic (black traces) and dendritic (blue traces) recordings show that the presynaptic SAC drives action potential firing, which back-propagated into the contralateral dendritic sub-tree. The lower trace shows the somato-dendritic attenuation of a sub-threshold SAC-evoked response. (**c**) Quantification of the sites of SAC-DSGC close appositions and spike-delay. Note that the majority of detected appositions were positioned in the dendritic subtree contralateral to the site of recording (151 ± 31 μm from the soma; *n*= 2). Recordings were made in the presence of GABAzine (10 μM).

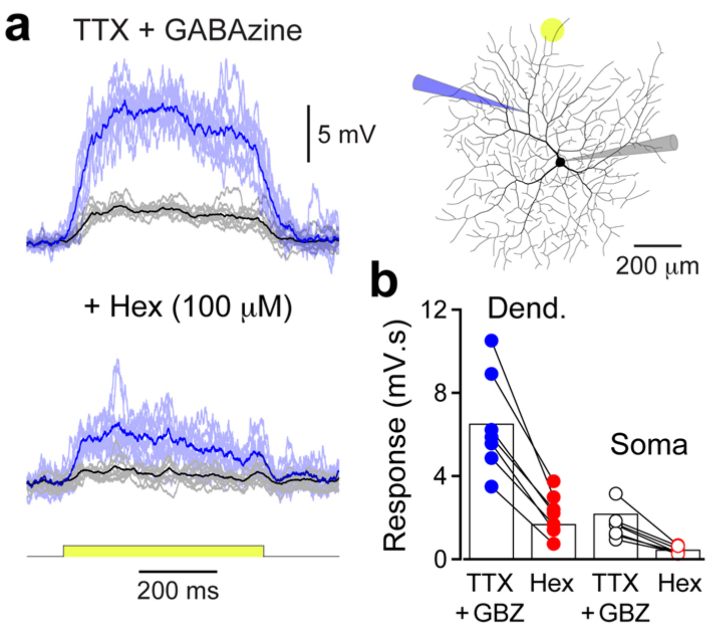

Supplementary Figure 10 Light-evoked SAC-mediated ACh release drives dendritic depolarization. (**a**) Antagonism of nAChRs (hexamethonium; Hex) powerfully attenuates light spot-evoked excitatory responses simultaneously recorded from somatic and dendritic sites. Responses were generated in the presence of tetrodotoxin (TTX; 1 µM) and GABAzine (10 µM). The lower trace shows the duration of the light spot stimuli. Note the pronounced dendro-somatic attenuation of light-evoked excitatory responses. The morphology of the ON-DSGC, placement of recording electrodes and position of the light spot stimuli are shown in the inset. (**b**) Quantification of the attenuation of the area of light spot-evoked excitatory responses by Hex. Dendritic recordings were 240 ± 12 µm from the soma.

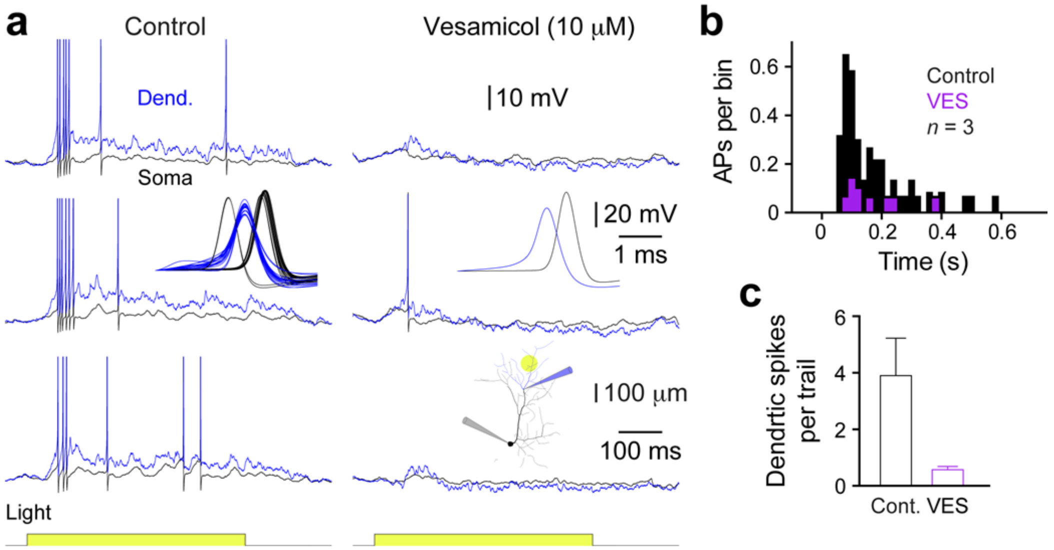

Supplementary Figure 11 Vesamicol powerfully attenuates light spot-evoked dendritic spike generation and neuronal output. (**a**) Simultaneous somatic (black traces) and dendritic (blue traces) recordings demonstrate that depletion of SAC-mediated ACh release (vesamicol; VES) attenuates light spot-evoked dendritic spike generation and consequential action potential (AP) firing. The morphology of the ON-DSGC, placement of recording electrodes and position of the light spot stimuli are shown in the inset. (**b**) Peri-stimulus time histograms of light spot-evoked (ON time= 0 to 0.5 s) AP output under the indicated conditions. (**c**) Dendritic spikes generated per trial under the indicated conditions (data represent mean ± s.e.m; control vs VES; P= 0.012, T= 9.0; n= 3).

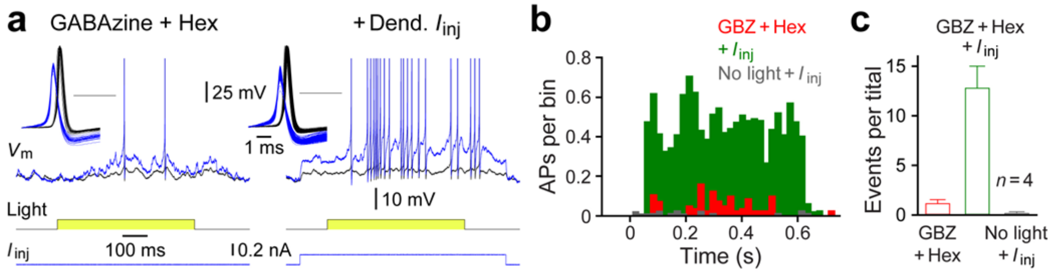

Supplementary Figure 12 Pairing of light spot stimuli with dendritic depolarization powerfully augments dendritic spike generation when SAC-mediated GABAergic and cholinergic signalling are blocked. (**a**) Pairing of light spot stimuli with sub-threshold dendritic depolarization dramatically increased dendritic spike generation when SAC-mediated excitation and inhibition were pharmacologically blocked (GABAzine (GBZ); 10 µM and hexamethonium (Hex); 100 µM). (**b**) Peri-stimulus time histogram of light spot-evoked (ON time 0 to 0.5 s) action potential (AP) output under the indicated conditions. (**c**) Number of large-amplitude dendritic spikes evoked by light spot-stimuli under the indicated conditions (data represent mean ± s.e.m).
